# Validation of machine learning models to detect neuropathologies

**DOI:** 10.1002/alz.088522

**Published:** 2025-01-03

**Authors:** Farooq Kamal, Cassandra Morrison, Michael Oliver, Mahsa Dadar

**Affiliations:** ^1^ Douglas Mental Health University Institute, McGill University, Montreal, QC Canada; ^2^ Carleton University, Ottawa, ON Canada; ^3^ Belmont University, Nashville, TN USA; ^4^ Douglas Mental Health University Institute, Montreal, QC Canada; ^5^ McGill University, Montreal, QC Canada

## Abstract

**Background:**

White matter hyperintensities (WMHs) are increasingly recognized for their role in cognitive decline and the progression of neurodegenerative conditions including Alzheimer’s disease (AD). Despite advances in imaging technologies, the exact contribution of WMHs to disease processes remains a subject of ongoing research. This study aims to apply machine learning approaches to determine critical features of AD‐related neuropathologies in vivo.

**Methods:**

A total of 65 participants (17 females, mean age = 79.0) from the Alzheimer’s Disease Neuroimaging Initiative (ADNI) were included. In the ADNI dataset, machine learning models were applied towards feature selection of MRI, clinical, and demographic data to identify the best performing set of variables that could predict neuropathology outcomes [e.g., Braak neurofibrillary tangle stage, Consortium to Establish a Registry for AD (CERAD) neuritic plaque, etc.]. The best‐performing neuropathology predictors using the top seven MRI, clinical, and demographic features were selected. For continuous measures, gradient boosting, bagging, support vector regression, and linear regression were implemented. For binary outcomes, logistic regression, gradient boosting, support vector machine, and bagging classifiers were utilized.

**Results:**

Four machine learning models applying feature ranking methods using similar information criteria consistently ranked WMHs as important features in predicting all neuropathology measures. In the ADNI dataset, prediction accuracy was highest for Braak stage, CERAD neuritic palques, and diffuse plaques (i.e., cross‐validated correlation between actual measures and predictions was above 0.8). The best‐performing model achieved over *r* = 0.85 correlation in predicting Braak.

**Conclusion:**

These results highlight the importance of WMHs as core features of AD and the benefits of using machine learning models that incorporate WMH burden in predicting AD‐related neuropathologies. The use of machine learning may prove beneficial in early detection of AD.

